# Learning from women veterans who navigate invisible injuries, caregiving, and reintegration challenges

**DOI:** 10.1186/s12905-023-02815-0

**Published:** 2023-12-11

**Authors:** Nicholas A. Rattray, Diana Natividad, Katrina Spontak, Marina Kukla, Ai-Nghia L. Do, Leah Danson, Richard M. Frankel, Gala True

**Affiliations:** 1 VA HSR&D Center for Health Information and Communication, Roudebush Veterans Affairs Medical Center, Indianapolis, USA; 2 Regenstrief Institute, Inc, Indianapolis, IN USA; 3 Indiana University School of Medicine, Indianapolis, USA; 4 Department of Psychology, Indiana-University-Purdue University, Indianapolis, USA; 5 University of Indianapolis, Indianapolis, USA; 6 South Central MIRECC, Southeast Louisiana Veterans Health Care System, New Orleans, LA USA; 7 Section of Community and Population Medicine, Louisiana State University School of Medicine, New Orleans, LA USA

**Keywords:** Veterans, Qualitative research, Women’s health, Social support

## Abstract

**Background:**

As women comprise a greater proportion of military service members, there is growing recognition of how their experiences in the early phase of military to civilian transitions have an important influence on their health and reintegration outcomes. Qualitative accounts of women veterans can inform programs that support transitioning service members.

**Objectives:**

We examined narratives of civilian reintegration among women veterans to understand their experiences of adjusting to community life while coping with mental health challenges.

**Methods/Participants:**

We interviewed 16 post-911 era women who were within 5 years of separating from military service and developed a case study based on three participants.

**Main approach:**

Interviews were audio-recorded and transcribed verbatim. Inductive thematic analysis was conducted to establish categories about reintegration. Immersion/crystallization techniques were used to identify exemplary cases that illustrated salient themes.

**Key results:**

Women veterans identified establishing a future career direction, drawing on social support, and navigating health care services as major factors influencing how they adjusted to civilian life. In addition, participants also highlighted the navigation of complex and intersecting identities (i.e., wife, mother, employee, friend, veteran, patient, etc.), further magnified by gender inequalities. These women performed emotional labor, which is often rendered invisible and oriented toward their family and loved ones, while simultaneously monitoring self-care activities. During the early period of reintegration, they described how they felt marginalized in terms of accessing healthcare compared to their military spouses and male veteran peers.

**Conclusions:**

Our case study suggests that there are key gaps in addressing healthcare and readjustment needs for women servicemembers, a high priority VA group, as they transition into post-military life. It is important to consider innovative ways to address specific needs of women in veteran-focused policies and programs.

**Supplementary Information:**

The online version contains supplementary material available at 10.1186/s12905-023-02815-0.

## Background

Military community reintegration (CR) has been defined as a return to participation in civilian life roles following discharge from military service and encompasses familial and social relationships, work, and other productive activities [1, 2]. Adjusting to civilian life can be more challenging for veterans with invisible injuries, a term that includes traumatic brain injuries and a range of mental health diagnoses. Such conditions are less visible than physical wounds and are prevalent among US veterans who have separated after 2001 [3]. Current evidence suggests that women are more likely than their male counterparts to have depression, anxiety, and issues with premature separation from military service, all factors which negatively impact CR [4, 5]. Women make up an increasing proportion of servicemembers and an even higher number of recruits, yet also report less satisfaction and shorter military careers than men [6, 7]. Women also have a high incidence of military sexual trauma (MST) [8–10] and enter the military with a higher rate of prior sexual abuse, [11] which is associated with an increased risk of post-traumatic stress disorder (PTSD) [12]. Studies have also shown that depression influences well-being and how women function within their household context [13, 14].

Among younger veterans, studies have shown that women report higher rates of service-connected mental health disability and healthcare utilization through the Department of Veterans Affairs (VA) than men of similar age [15, 16]. The increasing presence of women service members underscores the need to better understand how family life, particularly the challenges of providing care for children and military spouses, [17] and psychological health contribute to military to civilian integration and life after women’s military careers [18, 19].

The social needs of transitioning women veterans intersect with their experience in the military, as well as existing economic and social norms, such as prevailing family or gender roles [20, 21]. Recent research has shown that many veterans service organizations (VSOs) tend to focus on rank, branch, or other military characteristics to distinguish between veterans rather than issues of race, sexuality, or gender, [22] and that women do not typically feel welcomed at traditional VSOs [23]. Studies have also demonstrated that “universalist” health programs are, in practice, oriented toward men and lack gender sensitivity, which tends to marginalize women veterans in clinical treatment and discourage continuity in care [24–26]. In some studies, a limited number of women participants has led to concerns about statistical significance, which raises the issue of whether our current understanding of reintegration generalizes to women veterans [27, 28]. A few cross-sectional qualitative studies have examined how women view military to civilian transitions and access to health care [1, 29, 30]. Dichter and True [1] shed light on the trajectories of women veterans who experienced premature separation, while Williams et al. [30] emphasized how trauma could lead to a “tipping point” where women Veterans realized they required additional resources. Both studies highlight how women veterans face complex identity issues, including the need for tailored mental health [30].

We seek to fill a knowledge gap of the first few years of civilian reintegration among women veterans. Given the paucity of empirical studies, the study utilized a discovery-oriented approach using repeated interviews to explore practical suggestions about overlooked issues that women veterans face amidst readjustment to civilian life. These interviews examine the needs and challenges of women that might make it more difficult to engage them in programs designed to aid in navigating the transition from military to civilian life, as well as in health care to address their physical and mental health needs. Additionally, we evaluate the intersecting factors, roles, and identities (e.g. class, race, military rank/experience, family roles and responsibilities) that affect their lives as they transition [31] and how these socially meaningful characteristics operate in conjunction rather than independently and are intertwined rather than additive [32, 33].

We used a case study approach through a secondary analysis of a longitudinal observational study of veterans with invisible injuries, such as mental health issues, who have separated from military service. Our research questions centered on the unique reintegration experiences of women veterans and the factors that are the most challenging and difficult to overcome. Three cases of women veterans who also identify as mothers are highlighted, two of whom are also military spouses, to represent the intersecting roles women often carry with them from their service and continue to experience during the reintegration process.

## Methods

Institutional Research Board (IRB) approval was obtained from the VA medical center and its university affiliate. Participants were recruited from a midwestern VA medical center and its associated outpatient clinics. We completed a total of five interviews over 24 months with 16 women veterans as part of a longitudinal study examining the reintegration experiences of US military veterans within five years of separating from military service [34]. Appendix A outlines our methods using the “Consolidated criteria for reporting qualitative research” [35].

### Sample and recruitment

Veterans were eligible if they had an “invisible injury,” which was defined as a diagnosis of a mental or cognitive health disorder (including PTSD, traumatic brain injury, mood disorder, or anxiety disorder) confirmed in VA electronic health records. Participants were excluded if they had dementia or other significant cognitive impairment that would prevent giving informed consent. Eligible veterans were identified from electronic health records and mailed letters describing the study. Potential participants were contacted by phone to confirm eligibility and interest and to schedule a baseline study visit. As a result of protocol adaptation during the COVID-19 pandemic, we scheduled visits as either in-person, by phone, or using videoconferencing, depending upon participant preference. Recent literature has suggested that while security and ethical issues are important to consider, there are negligible differences between videoconference and in-person interviews [36, 37].

### Data collection

At each the baseline assessment, participants met with either the primary investigator, the research project manager, or one of two trained research assistants. After discussing the study aims, the interviewer obtained informed consent and HIPAA authorization, collected demographic information, and conducted a semi-structured interview lasting 60–90 min. We collected data at baseline, with follow-up assessments at 6, 12, 18, and 24 months. Participants received a $25 gift card for each completed interview. Interview topics included challenges with reintegration, identities and roles, social support, benefits, and mental and physical health issues and treatment. Follow-up interviews focused on health updates, the home environment, and ongoing readjustment challenges (See Appendix B for baseline, 6-month, 12-month, 18-monht, and 24-month interview guides).

### Data analysis

**Fig. 1:**
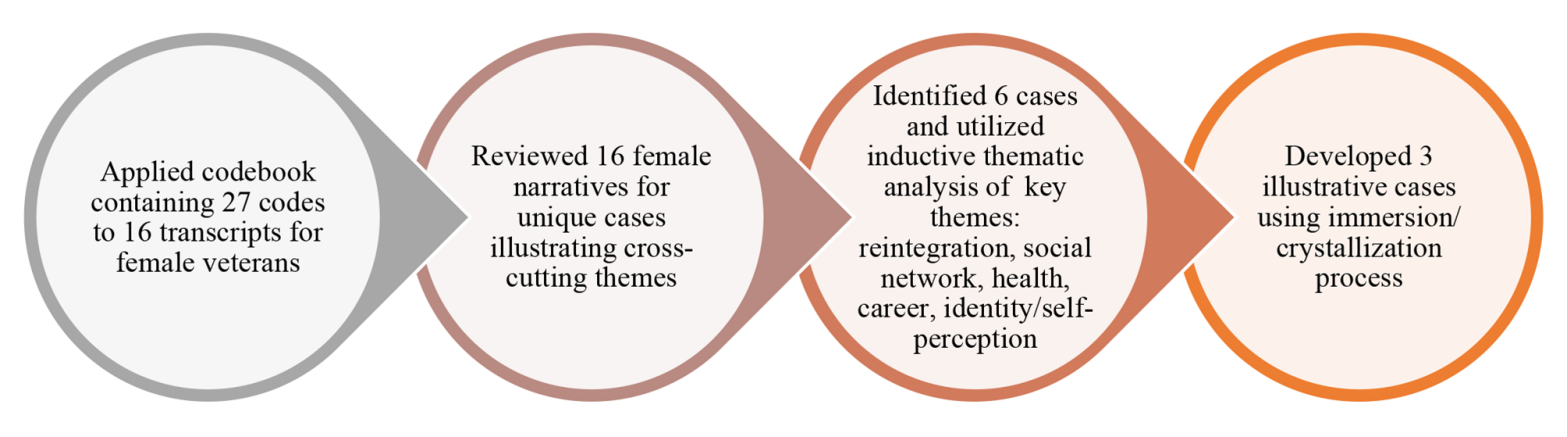
Process for Identifying Key Cases

**Fig. 2:**
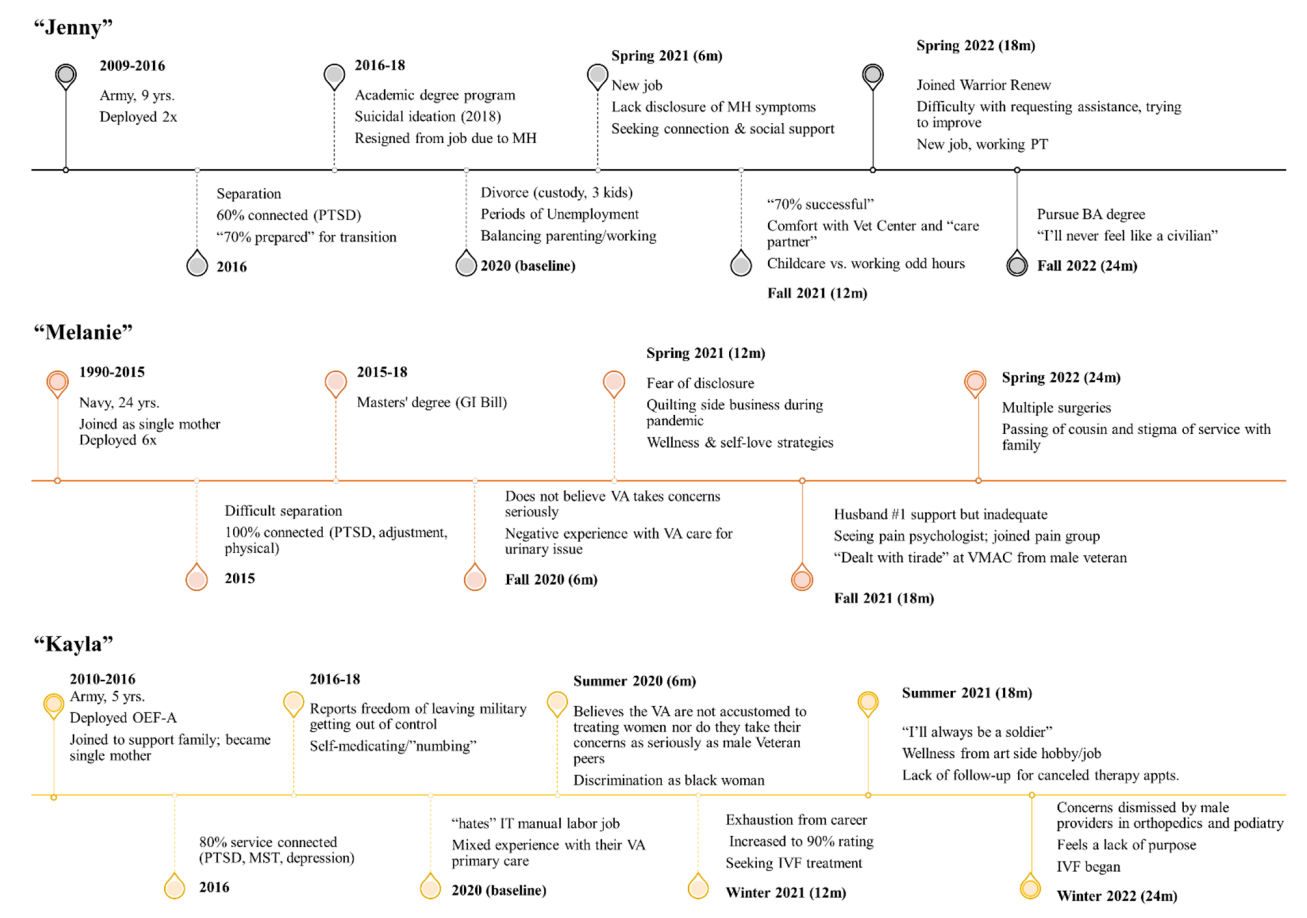
Timeline

Using a grounded theory approach, [38] a team of four analysts participated in a series of coding cycles to develop a codebook and code the interview transcripts (see Fig. 1). The first cycle of coding involved “open-coding” to identify key themes. After developing a codebook with 28 codes, we individually coded four transcripts and arrived at consensus to confirm the codebook reliability and align code definitions/inclusion criteria. The second cycle coding involved applying codes to each transcript. For this case study analysis, [39] baseline transcripts for all the participants were coded. Based on saliency and frequency of key codes from the second cycle coding, we extracted excerpts (i.e., career, identity/self-perception, family/social network, gender, health and healthcare/treatment, social network, reintegration). These excerpts addressed how women felt invisible as patients, the importance of social support, and the burden of caregiving duties. We identified six potential cases through a series of group meetings, which were then analyzed using immersion/crystallization, [40] a qualitative research approach that involves multiple reviews of data (immersion) with limited prior assumptions about discovery until consensus is reached (crystallization). Finally, we selected three exemplary cases that best illustrated cross-cutting themes and reviewed coded excerpts from their follow-up interviews (6, 12, 18, and 24 months). Figure 2 depicts the timeline of the three cases examined. As described in our COREQ (Appendix A), we used case summaries, member checking, and analytic memos to ensure the trustworthiness [41] of our findings.

## Results

Of the 16 study participants, we highlight three cases—Jenny, Melanie, and Kayla (pseudonyms)—whose differing reintegration experiences are defined by managing multiple roles, social well-being, financial challenges, gender-based career barriers, and discrepancies in their healthcare experiences compared with their male counterparts, which were salient themes across the broader sample of 16 participants. Each case highlights how female veterans balance the challenges of making sense of their identity and worth while navigating health care systems, achieving purposeful professional work, and building emotionally supportive relationships (see Table 1).

**Table 1:** Summary of key cases

Case	Demographic	Career	Healthcare	Family/ Social	Reintegration
*Jenny*	Early 30s, White, Army 9 years, 2 OEF deployments	Worked in the banking industry, quit due to mental health and to be present with children; works part-time and part- time school (as of 24 m)	PTSD, Depression, Anxiety, MST; Receives mental health care through Vet Center; assigned to male providers despite request for female only providers in response to dermatology clinic experience, reports fear of burden & retaliation	Divorced, single mother raising 3 children; Best friend/neighbor is primary support; some contact with mother and grandmother but report “they’re toxic”	Moderate improvement; mental health is improved slightly since baseline; limited social support; continued issues with disclosure and asking for help; balancing childcare, part-time job, and school
*Melanie*	50s, White, Retired Navy after 24 years, combat deployment	Lone female in security agency; has three master’s degrees	PTSD, MST; doesn’t seek out mental health care due to stigma but receives physical health care at the VA; experiences challenges with VA providers’ lack of knowledge of how to treat women-specific conditions; discusses necessity of self-advocacy in her care	Initial report of wide social network, married to a veteran with 3 adult sons from previous marriage; Quality of social connections weaker than initially perceived, limited support from husband (24 m)	no current issues with mental health but utilizes self-care in form of quilting, essential oils, etc. Demonstrated high success until final interview; more limited & less quality social support, challenges connecting with sons; barrier to access mental health due to career; 7 years post-separation & feels she is “still a work in progress”
*Kayla*	Early 30s, Latina/ African American, Army 5 years, deployed to OEF-A	Lone female in telecommunications industry performing manual labor; has some college education	Significant physical injuries, PTSD, fertility issues; seeks out health care at the VA but reports lack of knowledge and availability of women’s healthcare	Married to a Veteran but lacks support; Has one child from previous marriage and experiences fertility challenges	Overall minimal improvement, “it is what it is” mentality; moderate mental health improvements in therapy; financial stressors & physical demands of job impact quality of life

^a^ Career: descriptive information from Education and Work/Career codes

^b^ Healthcare: perspectives with providers, medication, treatment from Health Services code

^c^ Family/Social: content about social support, family, and friends from Social Network code and subcodes

^d^ Reintegration: content from Early Transition, Identity/Self-Perceptions and Reintegration codes

### Jenny: advocating for her social, mental, and career well-being

Jenny, who identified as white and in her 30s, served 9 years in the Army with two deployments, one to Iraq and one to Kuwait. When we first interviewed Jenny, she was recently separated from her husband and had custody of her three children, each under 10 years old. Jenny was diagnosed with chronic PTSD, and described dealing with anxiety and depression that led to seeking mental health care at the VA. Despite having numerous responsibilities as a single, working mother, she struggled with asking for help and often managed on her own, a mentality instilled in the military.

In the initial interviews Jenny worked in the banking industry but worked odd hours, which coincided with caring for her children after school hours. At the 12 and 18-month interviews, she described how improved mental health would enable her to return to her degree program. However, she describes lacking “grit” and not being able to advocate for herself to get a job commensurate with her worth. In subsequent interviews, Jenny transitioned to a part-time position with a veteran organization, which allowed her to balance her children’s needs after school along with her financial needs and career goals and helped her achieve a sense of purpose. At the final interview, Jenny had begun courses toward a business degree, but was again struggling with balancing work, school, and caring for her children with limited support.

Jenny described just a few supports that she felt comfortable with a neighbor she was close to, her veteran friend, and her therapist. At times, her grandmother and mother were helpful, but she preferred to keep her children away from them because she views them as “toxic.” Despite participating in a female veteran group, she felt lonely and struggled to advocate for herself.

Jenny first reached out for mental health care after having suicidal thoughts, but initially had a negative experience with her VA counselor who made her feel like a burden. She settled on seeing a Vet Center counselor rather than a traditional VA mental health provider because she could attend weekly sessions. In a follow-up interview, Jenny explained her therapist retired and she was waiting to be assigned to a new provider before resuming sessions, which negatively impacted her mental health. Jenny has acceptable physical health care at the VA, but has had experienced numerous issues, such as an uncomfortable experience with a male physician at the VA. Instead of reporting his incident and advocating for herself, she chose to move her care to female providers and to avoid feeling like a burden as she had felt in the past with her VA care experiences. Yet, despite this request, Jenny continued to be assigned to male providers in specialty clinics that continued to leave her feeling uncomfortable. She expressed a fear of “being that veteran,” with accompanying consequences in her care if she advocates for herself. She experienced negative encounters with her primary care providers where her medical complaints were dismissed as not significant.

A year after the baseline interview, Jenny indicated that she had been “70% successful” with her reintegration process. In follow-up interviews, Jenny continued to struggle to balance childcare responsibilities, financial strain, and her own stress management. She pursued education with the goal to open her own business but reported difficulty juggling multiple roles.

### Melanie: “continuing the fight” through self-care and social wellness

Melanie, a white female in her early 50s, retired after 24 years in the Navy. She has a 100% disability rating from numerous physical injuries, has been diagnosed with chronic PTSD, and experienced sexual assault while in the military. She rarely discloses information about these invisible conditions because she fears her employer may react negatively to her mental health issues. Melanie reflected on strategies to combat stress that occurred during reintegration and suggested in initial interviews that she was “thriving” from these adaptations. Lessons Melanie took from her Naval career, especially as a mother who joined to support her three sons, have led to a sense of resilience in her early transition to civilian life.

Although Melanie valued her stable and lucrative job, she discussed mistreatment from male co-workers that paralleled her experience in the Navy. Melanie was the lone female at her job, and she reflected on how she coped by adjusting her perspective: “I’ve had worse. I can do this for another 5 years.” At a follow-up interview, Melanie contrasted her experience with women in the Navy with her civilian workplace: “you don’t find a whole lot of queen bees in the military like you do in the civilian sector—by queen bee I’m just talking about that woman that makes it to the top and then [is] not willing to help other women.” Melanie later changed jobs and reported improved workplace dynamics, but she worried about how her security clearance could be impacted by disclosing any mental health conditions or issues.

In initial interviews, Melanie reported a wide social network including family, friends, and acquaintances, all of whom she viewed as integral to her well-being. Melanie had the strongest network of other cases in this sample women. She purposefully sought out such allies and viewed this type of support as integral to her success: “I was a pretty tough bitch … to have these guys think that because I’m in civilian clothes and my hair is down or whatever that it’s okay to screw with me. No, it’s not…part of the reason if any of my girls [fellow veterans] came to me and said something was happening that shouldn’t be- I fought way harder for them probably because no one fought for me.” In later interviews, Melanie reported a striking shift in her social relationships, realizing that many of her relationships were not as reciprocal and strong as she previously thought. Partially as a result of the COVID-19 pandemic, she lost contact with key supporters within her family.

Melanie utilized primary care but had poor experiences with mental health, women’s health providers, and her general VA care. She explained, “I don’t have any ongoing mental health care–there’s still a stigma attached to it—Much like the one I encountered when I was seeking mental health and… was basically persecuted for it. I also have a very high security clearance, so I use a lot of coping mechanisms— not like I go drink —it’s more like oils.” Melanie described how natural oils and quilting helped her cope with stress and past trauma. While she recognized that there may not be sufficient demand for a full-time gynecology department or a mammogram machine, she was dissatisfied with the lack of knowledge among male providers and access to women-specific healthcare within the VA, such as when providers were unable to insert a catheter after surgery which led to an emergent situation: “they almost immediately want to outsource you because you’re a female.” Furthermore, Melanie has had multiple surgeries with the VA, including one procedure where sutures that remained in her body led to significant pain.

At the one-year interview, Melanie discussed her “I love me” wall at home, which has memorabilia and awards from military service. The wall made her think of a recent incident where a veteran verbally assaulted her following an encounter in the parking lot when he saw that she was wearing a hat indicating that she was a chief petty officer. She recalled having to call security when the man escalated the situation. Melanie was especially frustrated about having to deal with such treatment at the VA hospital, directly from a fellow veteran.

Melanie’s transition from military life initially appeared positive due to her proactive involvement with social supports, resilience at work, and ability to adapt to different situations; all three conditions appear to be linked to her as a role caretaker. However, the final interview demonstrated that Melanie was struggling more with transition to civilian life than it initially appeared, lacked strong social support, and was burnt out from being the “tough cookie.” At the start of the COVID-19 pandemic, Melanie struggled with not being able to see friends and family who lived far away. While she cultivated a strong relationship with her neighbor during this period, once restrictions were lifted, Melanie began to realize the lack of quality and effort in some of her relationships. In addition, her cousin died, who was her best friend, and did not receive adequate support from her husband throughout her mourning. While she attended therapy in the past, at the time of the final interview she was no longer in treatment due to her job and relied on quilting and other holistic methods to help with stress. Melanie expressed—seven years post-separation—that reintegration would continue to be a daily struggle where she was unsure if she would ever be fully assimilated to civilian life.

### Kayla: finding support in a male-dominated career and healthcare system

Kayla was originally from the West Coast and identifies as first-generation Latina and African American. She joined the Army to support her daughter and ex-husband. In conjunction with pre-military trauma, Kayla experienced sexual assault while in the military which continued to affect her mental health. After separating from the Army, she struggled to address her physical ailments and fertility issues and find time for her family, while also having a physically and mentally demanding job in a male-dominated career. The intersecting effects of limited social support, health issues, and work stress contributed to her depression and anxiety.

Kayla’s work environment affected her health and family negatively. She felt constrained by financial stress and the need to support her family, factors that had weighed on her prior to military service. Her job was physically demanding, and she felt forced to tolerate inappropriate comments from her co-workers. She was resistant to pressure to switch to a “traditionally feminine” career, which would require financial sacrifice and further education. At her follow-ups, she noted feeling desensitized to her uncomfortable work environment because of her military experiences. As the lone female at work, her needs as a mother were overlooked: “I went right back into being in a male-dominated industry…I was like hey, we’re going to try and have a baby. What do you guys have for maternity leave? …They don’t have an actual maternity program put in place because I work in a place…Because I work in a male-dominated industry, and they don’t have to deal with it.” In follow-up interviews, she added that her job provided limited time off and she worried for her job security as her employer intended to “put her on disability” when she became pregnant. Kayla recounted how health providers had encouraged her to switch professions to improve her physical health, which she felt was unrealistic.

Kayla described difficulty disclosing her mental and physical health issues to her husband who is also a veteran she served with. However, she did not fully disclose daily struggles to her husband at times, knowing coworkers’ comments would upset him. Kayla described how she lacked co-workers or friends to “vent with” and felt unable to turn to her natal family. In describing her first-generation immigrant family, she struggled with living far from them and having to rely on her husband rather than her parents: “some people, they just go back home—they know that ‘I’m going to have my mom and dad around.’ If something happens, I’ve got backup. I didn’t have that.” Kayla described how her post-traumatic stress was exacerbated by her role as a caretaker for her veteran husband, leading to occasional suicidal ideation: “If I have a day where I feel like that I might hurt myself, I don’t really have anybody other than my husband that I feel like that I can talk to, and even with him…I don’t want to talk to him necessarily because I don’t want it to affect him.”

Kayla reported negative experiences with treatment for physical injuries, mental health care, and in seeking fertility treatment. She perceived that her providers disregard her physical complaints and spoke to her in a condescending manner rather than addressing her health issues holistically. She offered the rhetorical question: “So what are we going to do so that I can feel better so that I can make money to survive? That’s not their overall goal—it’s to say I marked this box off, and she’s good to go on my end.” Lengthy or delayed healthcare appointments forced her to prioritize work over clinic visits. Kayla reported difficulty with obtaining treatment through the VA for her fertility issues, which she viewed as directly related to her military service. While she liked one of her key therapists, Kayla rarely addressed her mental health issues directly, saying, “Most of the time…it’s easier to talk about what you want to talk about and not deal [with] what you need to talk about.” She was unable to meet with her therapist more often than every 6 weeks, and later switched to a new therapist whom she was less satisfied with and continued to have difficulty scheduling appointments. In her final interview, Kayla explicitly expressed her perspective that her care at the VA was poor because she is young, female, and African American—all factors that affect how she has been treated. Nearly six years after separation, Kayla did not feel readjusted to civilian life, but rather found similarities between her current situation and the same barriers she felt in her male-dominated military career. She struggled to balance her family’s needs and her own healthcare needs with pressures to “act tough and masculine” at work. Kayla described poor experiences receiving emergency care at the VA as a female and reflected on how she received worse care than her husband. She turned to alternative methods to remedy her pain from her physical injuries and avoid surgery and medication such as massages, dry needling, chiropractic care, and Epsom salt baths. She likewise found it beneficial to create an art studio to help with mental health issues. Despite Kayla’s attempts to improve her situation, over her two years in the study the combination of work-related challenges, inadequate social support, and healthcare barriers stymied her readjustment.

## Discussion

As women comprise an increasing proportion of the Veteran population, there is a pressing need to better understand their experiences as they transition to civilian life. We used a case study approach to examine how three women with invisible injuries have reintegrated into post-military lives in the domains of career, social support, roles and identities, and healthcare. In their self-perception of community reintegration, it is apparent that transitioning into a post-military career is the highest priority due to financial concerns and the logistics of raising children, represented by Jenny and Kayla, while less obviously important, social support is a key challenge that impacts women veterans and their mental health, highlighted by Melanie’s experience. However desirable it is, social support may be difficult to attain in the context of challenges to work-life balance [42]. Lastly, the three women call attention to inconsistency in gender sensitivity in VA health services [22, 26]. Furthermore, these cases illustrate barriers to access care and pressure for self-advocacy in response to feeling undervalued or dismissed by VA health practitioners.

We illustrated the experiences of veterans who are mothers and are currently, or were previously, military spouses to highlight the interplay between the competing demands of work and home. Compared to male veterans, establishing a stable sense of self as a civilian requires a complex set of demands that include roles as wife, mother, employee, friend, veteran, and healthcare patient. Kayla and Jenny emphasized how VA healthcare has been developed to serve primarily male patients and is more broadly emblematic of employment sectors where women are often rendered invisible. These women performed emotional labor [43]—which is likewise undervalued—and oriented toward their family, while simultaneously monitoring self-care activities and personal needs. Melanie exemplifies how women frequently compare their reintegration experiences to their male veteran peers, and husbands, often finding that men fared better. At the same time, the resources they sought within their careers, social support, and healthcare were often tailored to serve their male peers [44]. Female veterans often experience dismissal of complaints and ignored requests, compounding their difficulties in quality of care. Finally, Melanie and Kayla described a constant battle to appear ‘tougher’ and ‘more worthy’ than their male veteran peers.

Building on momentum toward viewing gender as an analytic category in veteran research, this study foregrounds how women situate themselves in relation to their male veteran peers and within social hierarchies at home and at work [45, 46]. A consistent theme from women in our study was feeling the effects of “reverse culture shock,” or the experience of difficulty adjusting to life in US society after long military careers, but from their perspective as a woman [47, 48]. Although this tendency has been identified for veterans in general, less attention has been given to how women deal with loss of camaraderie after working closely with men during military service; it could be that prevailing gender norms make such close relationships more unlikely to occur in civilian contexts, particular for women with partners [29]. While hundreds of programs have been developed to support veterans, evidence suggests that women veterans report persistent barriers to utilization [49]. Recent transition programs specifically focused on upstream prevention of mental health issues during the early transition period [30, 50, 51] should address what causes women to feel marginalized in health care, account for common caregiving responsibilities that compound barriers to healthy reintegration, and be trauma-specific [30].

The strength of the discovery-oriented approach in this qualitative study is the emphasis on intersecting themes shared by women veterans by drawing on their own perceptions, following standards for case study analysis in health services research [47, 52]. Another strength of the present study is the longitudinal approach which allowed increased likelihood of disclosure and comfort during interviews, demonstrated by the often increase in disclosure in each interview compared to previous encounters. We highlighted only three veteran cases, drawn from a subsample of 16 women, which limit claims that can be made about the transferability [41] of our findings. The extent to which these findings are applicable to the larger population of post-911 women veterans is unknown.

## Conclusion

Delving into the narratives that women veterans offer as part of longitudinal qualitative studies can deepen our understanding of patient experiences among this growing veteran population. While the health needs of women have long been a priority in the VHA, a deeper understanding of how women veterans experience multiple contexts, including healthcare, can aid in integrating innovative ways to address specific needs of women in veteran-focused policies and programs.

### Electronic supplementary material

Below is the link to the electronic supplementary material.

Supplementary Material 1

Supplementary Material 2

## Data Availability

These data must remain on Department of Veterans Affairs servers; investigators interested in working with these data are encouraged to contact the corresponding author.

## References

[1] Dichter ME, True G (2014). This is the story of why my military Career Ended before it should have: premature separation from Military Service among U.S. Women veterans. Affilia.

[2] Elnitsky CA, Blevins CL, Fisher MP, Magruder K (2017). Military service member and veteran reintegration: a critical review and adapted ecological model. Am J Orthopsychiatry.

[3] Tanielian TL, Jaycox L (2008). Invisible wounds of War: psychological and cognitive injuries, their consequences, and services to assist recovery.

[4] Flodgren G, Parmelli E, Doumit G, Gattellari M, O’Brien MA, Grimshaw J et al. Local opinion leaders: effects on professional practice and health care outcomes. Cochrane Database Syst Rev. 2011;(8):CD000125. 10.1002/14651858.CD000125.pub4 PMC4172331 21833939

[5] Vogt DS, Tyrell FA, Bramande EA, Nillni YI, Taverna EC, Finley EP (2020). U.S. military veterans’ Health and Well-being in the First Year after Service. Am J Prev Med.

[6] Nuciari M, Caforio G, Nuciari M (2006). Women in the military. Handbook of the sociology of the military.

[7] Simon RJ. Women in the military. Transaction Publishers; 2001.

[8] Campbell R, Raja S (2005). The Sexual Assault and secondary victimization of female veterans: help-seeking experiences with military and civilian Social systems. Psychol Women Q.

[9] Suris A, Lind L (2008). Military sexual trauma: a review of prevalence and associated health consequences in veterans. Trauma Violence & Abuse.

[10] Sadler AG, Booth BM, Mengeling MA, Doebbeling BN (2004). Life span and repeated Violence against women during military service: effects on health status and outpatient utilization. J Womens Health (Larchmt).

[11] Lang AJ, Aarons GA, Gearity J, Laffaye C, Satz L, Dresselhaus TR (2008). Direct and indirect links between Childhood Maltreatment, Posttraumatic Stress Disorder, and women’s Health. Behav Med.

[12] Murdoch M, Polusny MA, Hodges J, Cowper D (2006). The Association between In-Service sexual Harassment and post-traumatic stress disorder among Department of Veterans affairs disability applicants. Mil Med.

[13] Vogt D, King MW, Borowski S, Finley EP, Perkins DF, Copeland LA (2021). Identifying factors that contribute to military veterans’ post-military well-being. Appl Psychol Health Well Being.

[14] Smith BN, Taverna EC, Fox AB, Schnurr PP, Matteo RA, Vogt D (2017). The role of PTSD, Depression, and Alcohol Misuse Symptom Severity in linking Deployment Stressor exposure and Post-military Work and Family outcomes in male and female veterans. Clin Psychol Sci.

[15] Maynard C, Nelson K, Fihn SD (2019). Characteristics of younger women veterans with service connected disabilities. Heliyon.

[16] Amara J, Iverson KM, Krengel M, Pogoda TK, Hendricks A (2014). Anticipating the traumatic brain injury-related health care needs of women veterans after the Department of Defense change in combat assignment policy. Women’s Health Issues: Official Publication of the Jacobs Institute of Women’s Health.

[17] LaVela SL, Etingen B, Pape TL-B (2013). Caregiving experiences and health conditions of women veteran and non-veteran caregivers. Women’s Health Issues.

[18] Southwell KH, MacDermid Wadsworth SM (2016). The many faces of military families: unique features of the lives of female service members. Mil Med.

[19] Sherman MD, Larsen J, Borden LM (2015). Broadening the focus in supporting reintegrating Iraq and Afghanistan veterans: six key domains of functioning. Prof Psychology: Res Pract.

[20] Enloe CH. Nimo’s war, Emma’s war: Making feminist sense of the Iraq war. Univ of California Press; 2010.

[21] Segal MW (1995). Women’s military roles cross-nationally: past, Present, and Future. Gend Soc.

[22] Ahlin EM, Douds AS (2018). Many shades of Green: Assessing Awareness of Differences in Mental Health Care needs among subpopulations of military veterans. Int J Offender Ther Comp Criminol.

[23] Thomas KH, Haring EL, McDaniel J, Fletcher KL, Albright DL. Belonging and support: Women veterans’ perceptions of veteran service organizations. Journal of Veterans Studies. 2017;2(2).

[24] Cheney AM, Dunn A, Booth BM, Frith L, Curran GM, THE INTERSECTIONS OF GENDER AND POWER IN WOMEN VETERANS’EXPERIENCES OF SUBSTANCE USE AND VA CARE (2013). Annals of Anthropological Practice.

[25] Than CT, Washington DL, Vogt D, Chuang E, Needleman J, Canelo I et al. Discontinuity of Women Veterans’ Care in Patient-Centered Medical Homes: Does Workforce Gender Sensitivity Matter? Women’s Health Issues. 2021. 10.1016/j.whi.2021.11.008. 10.1016/j.whi.2021.11.008 34930639

[26] Huynh-Hohnbaum A-LT, Damron-Rodriguez J, Washington DL, Villa V, Harada N (2003). Exploring the diversity of women veterans’ identity to improve the delivery of veterans’ health services. Affilia.

[27] Kleykamp MA, College (2006). Jobs, or the military? Enlistment during a time of War. Soc Sci Q.

[28] Sayer N, Noorbaloochi S, Frazier P, Carlson K, Gravely A, Murdoch M. Reintegration problems and treatment interests among Iraq and Afghanistan combat veterans receiving VA medical care. Psychiatric services (Washington, D.C.). 2010;61(6):589–97. 10.1176/ps.2010.61.6.589 20513682

[29] Burkhart L, Hogan N (2015). Being a female veteran: a grounded theory of coping with transitions. Social work in Mental Health.

[30] Williams L, Pavlish C, Maliski S, Washington D (2018). Clearing away past wreckage: a Constructivist grounded theory of identity and Mental Health Access by Female Veterans. Adv Nurs Sci.

[31] Sasson-Levy O. Ethnicity and Gender in Militaries: An Intersectional Analysis. 2017. p. 125 – 43.

[32] Cole ER (2009). Intersectionality and research in psychology. Am Psychol.

[33] Warner DF, Brown TH (2011). Understanding how race/ethnicity and gender define age-trajectories of disability: an intersectionality approach. Soc Sci Med.

[34] Rattray N, Flanagan M, Salyers M, Natividad D, Do A-N, Frankel R et al. The Association Between Reintegration, Perceptions of Health and Flourishing During Transition from Military to Civilian Life Among Veterans with Invisible Injuries. 2023.

[35] Tong A, Sainsbury P, Craig J (2007). Consolidated criteria for reporting qualitative research (COREQ): a 32-item checklist for interviews and focus groups. Int J Qual Health care: J Int Soc Qual Health Care / ISQua.

[36] Archibald MM, Ambagtsheer RC, Casey MG, Lawless M (2019). Using zoom videoconferencing for qualitative data Collection: perceptions and experiences of researchers and participants. Int J Qualitative Methods.

[37] Buck KD, Roe D, Yanos P, Buck B, Fogley RL, Grant M (2013). Challenges to assisting with the recovery of personal identity and wellness for persons with serious mental Illness: considerations for mental health professionals. Psychosis.

[38] Charmaz K (2006). Constructing grounded theory: a practical guide through qualitative analysis.

[39] Yin RK. Case study research and applications: design and methods. Sage publications; 2017.

[40] Borkan JM, Crabtree BF, Miller WL (1999). Immersion/crystallization. Doing qualitative research.

[41] Lincoln YS, Guba EG. Naturalistic inquiry: sage; 1985.

[42] Rattray NA, True G, Natividad DM, Salyers MP, Frankel RM, Kukla M (2019). The long and winding road to postsecondary education for U.S. veterans with invisible injuries. Psychiatr Rehabil J.

[43] Abraham TH, Ono SS, Moriarty H, Winter L, Bender RE, Facundo R (2021). Revealing the invisible emotion work of caregivers: a Photovoice Exploration of Informal Care provided by Family caregivers for Post-9/11 Veterans with traumatic brain injuries. J Head Trauma Rehabil.

[44] Atherton S (2009). Domesticating military masculinities: home, performance and the negotiation of identity. Social & Cultural Geography.

[45] Eichler M (2017). Add female veterans and stir? A Feminist Perspective on Gendering Veterans Research. Armed Forces & Society.

[46] Higate P (2003). Military masculinities: identity and the state.

[47] Koenig CJ, Maguen S, Monroy JD, Mayott L, Seal KH (2014). Facilitating culture-centered communication between health care providers and veterans transitioning from military deployment to civilian life. Patient Educ Couns.

[48] Bergman BP, Burdett HJ, Greenberg N (2014). Service Life and Beyond – Institution or Culture?. RUSI J.

[49] Stecker T, Fortney J (2011). Barriers to mental health treatment engagement among veterans. Caring for veterans with deployment-related stress disorders.

[50] Sokol Y, Gromatsky M, Edwards ER, Greene AL, Geraci JC, Harris RE (2021). The deadly gap: understanding Suicide among veterans transitioning out of the military. Psychiatry Res.

[51] Geraci JC, Mobbs M, Edwards ER, Doerries B, Armstrong N, Porcarelli R (2020). Expanded roles and recommendations for stakeholders to successfully reintegrate modern warriors and mitigate Suicide risk. Front Psychol.

[52] Zamora KA, Abraham TH, Koenig CJ, Hill CC, Pyne JM, Seal KH. Using an adapted Case Study Approach to understand rural veteran experiences in Patient Engagement and patient-centered Care Research. Qualitative Res Med Healthc. 2020;4(2). 10.4081/qrmh.2020.8977.

