# Supplementary material for: Learning from women veterans who navigate invisible injuries, caregiving, and reintegration challenges
Source: BMC Womens Health. 2023 Dec 11;23:665. doi: 10.1186/s12905-023-02815-0 (PMC10714493; doi:10.1186/s12905-023-02815-0)
Supplement: Supplementary file 1 — Supplementary Material 1 [file 12905_2023_2815_MOESM1_ESM.docx]

**Supplementary Materials**

**S1 Table. Consolidated criteria for reporting qualitative studies (COREQ), 32-item checklist (Tong et al., 2007)**

| **No. Item** | **Guide questions/description** | **Reported on Page #** |
| --- | --- | --- |
| **Domain 1: Research team and reﬂexivity** | | |
| *Personal Characteristics* |  |  |
| 1. Interviewer/facilitator | NAR, DN, and AD conducted interviews. | Methods |
| 2. Credentials | NAR and GT have PhDs in Anthropology; MK has a PhD in Psychology; RMF has a PhD in Sociology; DN has an MPH; KS has an MS in Psychology; AD has a BA in English; LD has a BA in International Studies. | Title page |
| 3. Occupation | Health services and rehabilitation researchers (NAR, DN, KS, AD, LD, RMF) and university professors (RMF, GT, NAR, MK). | Methods |
| 4. Gender | Female (5) and male (2). | Not Included |
| 5. Experience and training | NAR and GT are PhD-trained medical anthropologists. RMF is a PhD-trained sociologist. MK are PhD-trained clinical and research psychologists. DN is a research coordinator and qualitative analyst and has experience in participant recruitment and Masters’ level training in mixed methods. KS is a health service researcher and qualitative analyst and has Masters’ level training in mixed methods. LD is a graduate student in psychology. AD is a health service researcher and analyst. | Not Included |
| *Relationship with participants* |  |  |
| 6. Relationship established | Relationships were established during study recruitment with contact from DN, AD, and NR | Not Included |
| 7. Participant knowledge of the interviewer | Prior to interviews, NAR, AD and DN provided descriptions of the project goals and the purpose of the interviews, as well as a brief personal introduction that included the motivation for the research, experience working with military Veterans, and professional training. | Not Included |
| 8. Interviewer characteristics | None | Not Included |
| **Domain 2: study design** | | |
| *Theoretical framework* |  |  |
| 9. Methodological orientation and Theory | Analysis of interview data followed a three-step, inductive approach that is consistent with grounded theory (Charmaz 2006). Deeper analysis of individual cases utilized an immersion/crystallization (Borkan, 1999), a qualitative research approach that involves multiple reviews of data (immersion) with limited prior assumptions about discovery until consensus is reached (crystallization). | The approach is described in the qualitative analysis section. |
| *Participant selection* |  |  |
| 10. Sampling | We recruited 16 women veterans and focused on 3 participants as cases. | Data collection section of Methods |
| 11. Method of approach | Clinician referral from three outpatient clinics (i.e., post deployment integrated health clinic, women’s health clinic, and mental health clinic) at a tertiary VA medical center. | Data collection section of Methods |
| 12. Sample size | Veterans with a diagnosed invisible injury who had separated from military service in the last 60 months. Veterans were eligible if they had an invisible injury, which was defined as a diagnosis of a mental health or cognitive health disorder (including PTSD, traumatic brain injury, mood disorder, or anxiety disorder) as documented in their VA or non-VA electronic health records. It is important to note that some conditions (e.g. anxiety) may have been preexisting and may have been exacerbated by military employment. Sixteen of the sample were women; 3 women were selected for in-depth case studies according to methods described above. | Sampling and Participant Selection |
| 13. Non-participation | After initial screening, several participants did not meet inclusion criteria. Other participants could not be scheduled for an in-person interview or did not appear for scheduled appointments after three follow-up calls. | Not included |
| *Setting* |  |  |
| 14. Setting of data collection | Interviews were conducted in a private meeting room at a tertiary VA medical center in an urban Midwestern city in the United States, over the phone, or using a video call. | Methods |
| 15. Presence of non-participants | No | N/A |
| 16. Description of sample | Participants who completed a baseline assessment and additional follow-up interviews included 16 female Veterans. Half of the participants served at least one deployment to Afghanistan, and 19% deployed to Iraq. Three branches of active duty were represented (Army, Navy, Air Force) including 1 participant involved in National Guard duty and 4 past inactive Reservists. The mean number of months since separation from the military was 34. The Veterans were representative of the current racial/ethnic demographics of the US military with 37.5% of participants who identified as non-white. The mean age was 36 years with a range of 24 to 53 years. | Table 1 |
| *Data collection* |  |  |
| 17. Interview guide | We used a semi-structured interview guide, including follow-up questions and prompts. The guide was pilot tested with two test participants using cognitive interviewing techniques. | Methods |
| 18. Repeat interviews | In the longitudinal study, participants are interviewed 5 times. This analysis is based on the complete data set with interviews conducted at baseline, 6 month, 12 month, 18 month, and 24 month increments. | Not included |
| 19. Audio/visual recording | Interviews were audio recorded. | Methods |
| 20. Field notes | Interview summaries written in the style of field notes were completed after each interview session and analyzed. These summaries were used to monitor emergent topics during a period of rapid analysis that preceded formal qualitative coding cycles. A data matrix of cases was constructed based on interviews summaries, and helped the team determined which participants had military spouses and/or represented critical themes as we selected cases. | Measures section of Methods |
| 21. Duration | Interview range = 30.19 minutes – 2 hours and 20 minutes  Average= 68.44 minutes | Not included |
| 22. Data saturation | We reached thematic saturation for all codes. | Qualitative data analysis section of Methods |
| 23. Transcripts returned | No | N/A |
| **Domain 3: analysis and ﬁndings** | | |
| *Data analysis* |  |  |
| 24. Number of data coders | Five | Qualitative data analysis section of Methods |
| 25. Description of the coding tree | We do not include a coding tree. Instead, we provide a general description of the coding process and codebook. | Qualitative data analysis section of Methods |
| 26. Derivation of themes | Themes emerged after coding the interviews through discussions among all three coders of the coding experience, their perceptions of participants’ reintegration success, field notes, case comparisons, and analytical memos. | Measures section and Qualitative data analysis section of Methods |
| 27. Software | QSR NVivo 12 | Qualitative data analysis section of Methods |
| 28. Participant checking | The parent study is longitudinal. Member checking occurs in follow-up interviews (6, 12, 18, 24 months). | N/A |
| *Reporting* |  |  |
| 29. Quotations presented | Participant quotations were presented to illustrate the findings. | Qualitative analysis section of Results |
| 30. Data and ﬁndings consistent | There is consistency between the data presented and the findings. | Qualitative analysis section Results and Discussion |
| 31. Clarity of major themes | Major themes are identified in subsections of each case. We found several major themes including, but not limited to, “work/education,” “social support,” “health services,” and “reintegration.”  Their implications are presented in the Discussion. | Qualitative analysis section Results and Discussion |
| 32. Clarity of minor themes | Minor themes are not included because we focused on three exemplary cases. | Qualitative analysis section Results and Discussion |
