# Supplementary material for: Learning from women veterans who navigate invisible injuries, caregiving, and reintegration challenges
Source: BMC Womens Health. 2023 Dec 11;23:665. doi: 10.1186/s12905-023-02815-0 (PMC10714493; doi:10.1186/s12905-023-02815-0)
Supplement: Supplementary file 2 — Supplementary Material 2 [file 12905_2023_2815_MOESM2_ESM.docx]

**VETERAN BASELINE INTERVIEW GUIDE**

| Participant ID: | Date: | Time: |
| --- | --- | --- |
| Interviewer(s): | | |

*This interview guide has two main parts.* ***Part B.1*** *is open-ended in nature and is intended to elicit the participant’s experience navigating the transition from military service to community life. One or more of the following “grand tour” questions will be used to gather the participant’s perspective on their own reintegration experience.*

**Part B.1**

- Can you describe your experience in finishing your military service and moving into civilian life?
- Did anyone follow-up with you after you left the military?
  - How did you feel about receiving (or not receiving) these follow-ups after separation?
- Can you guide me through the path that you’ve taken since leaving the military?
- In your own words, can you tell me about any health issues, physical or mental, that have played a role in your experiences since completing your military service?

*After the participant has discussed their overall transition or reintegration experiences,* ***Part B.2*** *of the interview explores additional themes. Which questions are covered depends on the extent to which topics were discussed in the initial narrative. Participants will be asked to answer with as much detail as possible.*

**Part B.2**

Reintegration or Transition Experiences

- Where were you stationed or where did you live during your military service? Can you describe your experiences living in these places and how they may have impacted your experiences after separating?
- **After separating, did you return to the city/town where you were living before you joined [the military branch]?**
  - **If no, where did you go instead and why? How did it impact your reintegration experiences, especially the earlier part of your transition?**
  - **If yes, why did you choose to go back there? How did it impact your reintegration experiences, especially the earlier part of your transition?**
  - **Would you call this a seamless transition or was the relocation disruptive?**
- What were some of your expectations or goals for when you returned? How are these expectations or goals being met or how have they changed?
- How would you describe your current housing situation?
- What is your day-to-day experience like as a civilian?
- How have your experiences been with your family, friends, colleagues, and other civilians since you returned? How have your relationships changed? How do they contribute to your reintegration experience?
- Do you think that gender shapes your experience in terms of reintegration? Does your race or ethnicity? Other forms of identity?
- **How successful do you think you’ve been in adjusting to life after separating from the military?**

Barriers and Facilitators

- What types of support have been important in your post-deployment experiences?
  - Can you describe any people, organizations, or other things that have been helpful in terms of reintegrating back into the community?
  - Can you think of any examples of experiences with specific people?
- Have you faced specific challenges or barriers? [Probe for different domains, such as family, social life, work, and leisure.]
  - What strategies have you used to deal with these challenges?
- Do you think your experience is typical of other veterans, or more uncommon?
- Are there any other factors that affect your success with reintegrating?

Health Issues and Invisible Injuries

- **Did you sustain any injuries that affected your transition? How have they impacted your transition?**
- **Have these injuries or conditions caused any limitations in your abilities? If yes, how so?**
- How comfortable are you with talking to people whom you have just met about injuries or your military experience? How do feel about disclosing information about [your health issue]?
- If you think about the health issue – it could be a physical or mental health condition, when did if you first experience difficulties for the first time?
  - If you went to receive services, either from a doctor or other health provider, can you describe that first visit or visits? Did you have any tests or treatments?
  - Did you ever have any health issues that you consider similar to this issue? In what way is it similar or different?
  - Has anyone else you’ve known had this type of issue or condition – in your family, or someone who you know closely? If yes, in what ways do you consider your experience similar or different?*
  - According to you, what caused your health issue? Are there any other causes that you think played a role? What happened inside your body that could explain your health issue/condition? Is there anything happening in your family, at work, or in your social life that could explain what is going on with this health issue/condition?*
  - Is your health issue linked to a specific event? Can you describe/explain how they are linked?*

Services and response to treatment

- During your initial visits to the doctor (healer) for your health issue, what did your doctor (healer) tell you that your health issue (HI) was?
- Did your doctor (healer) give you any treatment, medicine, or recommendations to follow? [List all] How are you dealing with each of these recommendations? Are you able to follow that treatment (or recommendation or medicine)?*
- What made that treatment work well? What made that treatment difficult to follow or work poorly? What treatments did you expect to receive for your (HP) that you did not receive? What other therapy, treatment, help or care have you sought out? What other therapy, treatment, help or care would you like to receive?*

Impact on life

- **How has your health issue (HI) changed the way you live? How has your HI changed the way you feel or think about yourself? How has your HI changed the way you look at life in general? How has your HI changed the way that others look at you?**
- What has helped you through this period in your life?
- How have your family or friends helped you through this difficult period of your life? How has your spiritual life, faith, or religious practice helped you go through this difficult period of your life?

Critical Junctures (In health or reintegration experiences)

- **Have there been any turning points or critical events that you can share?**
- How has your military experience defined who you are now?*
- What services or interventions have had a positive impact on your reintegration experiences?*
- What has been most or least unexpected about your transition?*
- Many people think about major milestones as typical parts of one’s life course. That can include finishing high school, starting a family, having a career, etc. Compared to what you might consider a normal life course or typical career, how do you think about your own experience? Have there been disruptions compared to what you expected?*

Moral Injury*

- **Can you describe some memories from your (combat) deployment(s) or overall military service that continue to cause you stress and inner conflict since you’ve returned?**
  - How do these memories impact you and your reintegration process?
  - Why do they continue to impact you in these ways?
  - What have you done to relieve the stress associated with these memories?

Social Support and Care Supporters

- What have been the greatest sources of support as you have moved from military to community life?
- What types of support do you receive from people in your family or wider support network?*
- Do you turn to specific people when you are struggling? How has this shifted, if at all, during your transition period? How would you compare the types support you received before or during your military service to your current sources of support?
- What types of support do you feel are missing or you wish you had to help you during your transition period?*
- Do you act as a source of support for any individuals? If you supported any individuals before your transition, how was your experience supporting them while in the military? How has your transition been impacted by having to support other individuals? What are some resources you wish you had to help you?*

Next Steps

- **What advice would you give to other Veterans with similar experience?**
- **Is there anything you would like to add that we haven’t spoken about so far?**
- If you don’t mind me asking, what are some of your future career plans?*
- Do you have suggestions in terms of improving services for returning Veterans? What services or interventions are still needed?*

**VETERAN 6-MONTH INTERVIEW GUIDE**

| Participant ID: | Date: | Time: |
| --- | --- | --- |
| Interviewer(s): | | |

*This interview guide has two main parts.* ***Part B.1*** *is open-ended in nature and is intended to elicit the participant’s experience navigating the transition from military service to community life. As much as possible, the participant will not be interrupted in this first part. One or more of the following “grand tour” questions will be used to gather the participant’s perspective on their own reintegration experience. These initial questions will be used as a marker to suggest some of the most important issues for the participant, to indicate where probing is needed, and to give the interviewer a chance to establish rapport.*

**Part B.1**

- Let’s start by touching base about your reintegration experience. You probably have some updates from the last time we spoke. Can you describe how things are going with respect to reintegration or adjustment to civilian life?
- Can you walk me through a typical day that you’ve had in the last few months? What is that like for you, and what is different compared to six months ago? What is your day-to-day experience like as a civilian?
- In thinking about health issues, physical or mental, can you talk about how they have played a role in your experiences since completing your military service or since we last spoke?

*After the participant has discussed their overall transition or reintegration experiences,* ***Part B.2*** *of the interview explores additional themes. Which questions are covered depends on the extent to which topics were discussed in the initial narrative. Participants will be asked to answer with as much detail as possible. Participants will be reminded that there are no right or wrong answers and that we’re asking for clarification and follow-up questions from the first part of the interview. In addition, the participant will be reminded about the anonymous, confidential, and voluntary aspects of the interview.*

**Part B.2**

Reintegration Experiences

- **Thinking about the last six months, have there been any changes in your expectations or goals? How are these expectations or goals being met?**
- **How have your experiences been with your family, friends, colleagues, and other civilians since you returned? How have your relationships changed? How do they contribute to your reintegration experience?**
- **We didn’t discuss much last time about aspects of your experience that might affect your transition. Do you think that gender shapes your experience in terms of reintegration? Does your race or ethnicity? Other forms of identity?**
- **If we asked a similar question that we asked at the baseline interview – “how successful do you think you’ve been in adjusting to life after separating from the military?” – how would you answer?**

Living Situation

- **Can we go back to places where you’ve lived either right before, right after or since separating. Where have you been stationed or lived during your military service? Can you describe your experiences living in these places and how they may impact your experiences after separating?**
- Did you return to the city/town where you were living before you joined [the military branch]?
  - If no, where did you go instead and why? How did it impact your reintegration experiences, especially the earlier part of your transition?
  - If yes, why did you choose to go back there? How did it impact your reintegration experiences, especially the earlier part of your transition?
- How would you describe your current housing situation?

Health Issues and Invisible Injuries

- **Returning to the topic of health issues, can we confirm what health issues you had or are currently experiencing? How have they impacted your transition?**
  - Have these injuries or conditions caused any limitations in your abilities? If yes, how so?
  - **How comfortable are you with talking to people whom you have not met about injuries or your military experience? How do feel about disclosing information about [X condition]?**
- **Regarding this health issue or condition, could you rate your current status ? Rate your current status as: 1) very much improved, 2) much improved, 3) minimally improved, 4) no change, 5) minimally worse, 6) much worse, or 7) very much worse**
- If you think about the health issue – it could be a physical or mental health condition, when did if you first experience difficulties for the first time?
  - If you went to receive services, either from a doctor or other health provider, can you describe that first visit or visits? Did you have any tests or treatments?
  - Has anyone else you know had this type of issue or condition – in your family, or someone who you know? If yes, in what ways do you consider your experience similar or different?
  - According to you, what caused your health issue? Are there any other causes that you think played a role?
  - Is your health issue linked to a specific event? Can you describe/explain how they are linked?
- **How has your health issue (HI) changed the way you live? How has your HI changed the way you feel or think about yourself? How has your HI changed the way you look at life in general? How has your HI changed the way that others look at you?**
  - What has helped you through this period in your life? How have your family or friends helped you through this difficult period of your life? How has your spiritual life, faith, or religious practice helped you go through this difficult period of your life?
- [If didn’t have or didn’t mention any mental or physical health care at baseline] **Have you sought VA or non-VA mental or physical health care since we last spoke? Why or why not?**
- [If did mention mental or physical health care at baseline] **Since we last spoke, how has the mental or physical care you received been?**
- **Please describe the process you went through to get a VA disability rating and to receive compensation and pension benefits.**

Moral Injury

- **Can you describe some memories from your (combat) deployment(s) or overall military service that continue to cause you stress and inner conflict since you’ve returned**?
  - How do these memories impact you and your reintegration process?
  - Why do they continue to impact you in these ways?
  - What have you done to relieve the stress associated with these memories?

Critical Junctures (In health or reintegration experiences)

- **Many people think about major milestones as typical parts of one’s life course. That can include finishing high school, starting a family, having a career, etc. Compared to what you might consider a normal life course or typical career, how do you view your own experience? Have there been disruptions compared to what you expected?**
- **Have there been any turning points or critical events that you can share?**
  - How has your military experience defined who you are now?*
  - What has been most or least unexpected about your transition?*

Care Partner & Social Support

- [If doesn’t have a care partner] Are there people or resources you’ve found helpful in the past 6 months? How have they helped you? For this question, you might think about the sociogram you drew for us at your first interview.
- [If has a care partner] Can you describe your relationship with your care partner? How has this person impacted you throughout your service, separation, and reintegration processes?
- How much contact did you have with your care partner while you were deployed?
- Are you and your care partner currently part of the VA’s paid care partner program?
  - If yes, how has this program been for both of you?
  - If not, do you plan to be part of this program in the future? Why or why not?

Next Steps

- What advice would you give to other Veterans with similar experience?
- Is there anything you would like to add that we haven’t spoken about so far?
- Do you have suggestions in terms of improving services for returning Veterans? What services or interventions are still needed?

**VETERAN 12-MONTH INTERVIEW GUIDE**

| Participant ID: | Date: | Time: |
| --- | --- | --- |
| Interviewer(s): | | |

*This interview guide has two main parts.* ***Part B.1*** *is open-ended in nature and is intended to elicit the participant’s experience navigating the transition from military service to community life. As much as possible, the participant will not be interrupted in this first part. One or more of the following “grand tour” questions will be used to gather the participant’s perspective on their own reintegration experience. These initial questions will be used as a marker to suggest some of the most important issues for the participant, to indicate where probing is needed, and to give the interviewer a chance to establish rapport.*

**Part B.1**

- Let’s start by touching base about your reintegration experience. You probably have some updates from the last time we spoke. Can you describe how things are going with respect to reintegration or adjustment to civilian life?
- Can you walk me through a typical day that you’ve had in the last few months? What is that like for you, and what is different compared to six or 12 months ago? What is your day-to-day experience like?
- In thinking about health issues, physical or mental, can you talk about how they have played a role in your daily experiences since we last spoke?

*After the participant has discussed their overall transition or reintegration experiences,* ***Part B.2*** *of the interview explores additional themes. Which questions are covered depends on the extent to which topics were discussed in the initial narrative. Participants will be asked to answer with as much detail as possible. Participants will be reminded that there are no right or wrong answers and that we’re asking for clarification and follow-up questions from the first part of the interview. In addition, the participant will be reminded about the anonymous, confidential, and voluntary aspects of the interview.*

**Part B.2**

Reintegration Experiences

- **Thinking about the last six months, have there been any changes in your expectations or goals? How are these expectations or goals being met? What about since we met a year ago?**
- **If we asked a similar question that we asked at the baseline interview – “how successful do you think you’ve been in adjusting to life after separating from the military?” – how would you answer?**
- **As you know, we ask questions about life purpose. Would you say there has been any changes with regard to how you think about purpose or meaning in your broader life goals?**

Living Situation

- **Can we go back to places where you’ve lived either right before, right after or since separating. Can you describe your experiences living in these places and how they may impact your experiences after separating?**
- How would you describe your current housing situation?

If home visit:

- **Can you describe your home in terms of where you spend most of your time?**

Meaningful Object (Picture, Artifact, Document, etc.)

- We asked you to consider an object or item that you consider meaningful. (If not prompted before interview, ask participant to find or think about an object or item that he or she considers to be meaningful.)
  - Which object did you choose, and why?

Health Issues and Invisible Injuries

- **Returning to the topic of health issues, can we confirm what health issues you had or are currently experiencing? How have they impacted your transition?**
  - Have these injuries or conditions caused any limitations in your abilities? If yes, how so?
  - How comfortable are you with talking to people whom you have not met about injuries or your military experience? How do feel about disclosing information about [X condition]?
- **Regarding this health issue or condition, could you rate your current status? Rate your current status as: 1) very much improved, 2) much improved, 3) minimally improved, 4) no change, 5) minimally worse, 6) much worse, or 7) very much worse**
- **Have there been any changes with your health treatment or health services that you use since we last spoke?**
- [If didn’t have or didn’t mention any mental or physical health care at baseline] **Have you sought VA or non-VA mental or physical health care since we last spoke? Why or why not?**
- [If did mention mental or physical health care at baseline] **Since we last spoke, how has the mental or physical care you received been?**
- **How has your health issue (HI) changed the way you live? How has your HI changed the way you feel or think about yourself? How has your HI changed the way you look at life in general? How has your HI changed the way that others look at you?**
  - What has helped you through this period in your life? How have your family or friends helped you through this difficult period of your life? How has your spiritual life, faith, or religious practice helped you go through this difficult period of your life?
- **Please describe the process you went through to get a VA disability rating and to receive compensation and pension benefits.**

Moral Injury

- **Can you describe some memories from your (combat) deployment(s) or overall military service that continue to cause you stress and inner conflict since you’ve returned**?
  - How do these memories impact you and your reintegration process?
  - Why do they continue to impact you in these ways?
  - What have you done to relieve the stress associated with these memories?

Critical Junctures (In health or reintegration experiences)

- **Many people think about major milestones as typical parts of one’s life course. That can include finishing high school, starting a family, having a career, etc. Compared to what you might consider a normal life course or typical career, how do you view your own experience? Have there been disruptions compared to what you expected?**
- **Have there been any turning points or critical events that you can share?**
  - How has your military experience defined who you are now?*
  - What has been most or least unexpected about your transition?*
- **We didn’t discuss much last time about aspects of your experience that might affect your transition. Do you think that gender shapes your experience in terms of reintegration? Does your race or ethnicity? Other forms of identity?**

Care Partner & Social Support

- **How have your experiences been with your family, friends, colleagues, and other civilians since you returned? How have your relationships changed? How do they contribute to your reintegration experience?**
- [If doesn’t have a care partner] Are there people or resources you’ve found helpful in the past 6 months? How have they helped you? For this question, you might think about the sociogram you drew for us at your first interview.
- [If has a care partner] Can you describe your relationship with your care partner? How has this person impacted you throughout your service, separation, and reintegration processes?
- How much contact did you have with your care partner while you were deployed?
- Are you and your care partner currently part of the VA’s paid care partner program?
  - If yes, how has this program been for both of you?
  - If not, do you plan to be part of this program in the future? Why or why not?

Interventions

- **Do you have suggestions in terms of improving services for returning Veterans? What services or interventions are still needed? What are some services you’ve recently utilized that have been most helpful during your reintegration in the past 6 months?**
- Some existing interventions or programs have been developed for veteran like you to support adjustment or reintegration. What sort of preferences would you have on the following:
  - In-person vs. virtual programs
  - Group vs. individual
  - Family or individual veterans
  - Working with a veteran peer
  - Talk therapy, writing exercises, etc.
- **Do you consider this study itself as a sort of program that affects your experiences with adjustment or reintegration?**
- **Has this study caused you to reflect more on your reintegration experience and as a result, caused you to take specific steps towards your goals to better reintegrate?**

Next Steps

- What advice would you give to other Veterans with similar experiences?
- Is there anything you would like to add that we haven’t spoken about so far?

**VETERAN 18-MONTH INTERVIEW GUIDE**

| Participant ID: | Date: | Time: |
| --- | --- | --- |
| Interviewer(s): | | |

*This interview guide has two main parts.* ***Part B.1*** *is open-ended in nature and is intended to elicit the participant’s experience navigating the transition from military service to community life. As much as possible, the participant will not be interrupted in this first part. One or more of the following “grand tour” questions will be used to gather the participant’s perspective on their own reintegration experience. These initial questions will be used as a marker to suggest some of the most important issues for the participant, to indicate where probing is needed, and to give the interviewer a chance to establish rapport.*

**Part B.1**

- Let’s start by touching base about your reintegration experience. You probably have some updates from the last time we spoke. Can you describe how things are going with respect to reintegration or adjustment to civilian life?
- Can you walk me through a typical day that you’ve had in the last few months? What is that like for you, and what is different compared to six or 12 months ago? What is your day-to-day experience like now?
- In thinking about health issues, physical or mental, can you talk about how they have played a role in your daily experiences since we last spoke?

*After the participant has discussed their overall transition or reintegration experiences,* ***Part B.2*** *of the interview explores additional themes. Which questions are covered depends on the extent to which topics were discussed in the initial narrative. Participants will be asked to answer with as much detail as possible. Participants will be reminded that there are no right or wrong answers and that we’re asking for clarification and follow-up questions from the first part of the interview. In addition, the participant will be reminded about the anonymous, confidential, and voluntary aspects of the interview.*

**Part B.2**

Reintegration Experiences

- **Thinking about the last six months, have there been any changes in your expectations or goals? How are these expectations or goals being met? What about since we met 18 months ago?**
- **How successful do you think you’ve been in adjusting to life after separating from the military since we last spoke?**
- Would you say there has been any changes with regard to how you think about purpose or meaning in your broader life goals?
- Has there been any change in your living arrangements or housing situation?

Health Issues and Invisible Injuries

- **Have there been any changes with your health treatment or health services that you use since we last spoke?**
- [If didn’t have or didn’t mention any mental or physical health care at baseline] **Have you sought VA or non-VA mental or physical health care since we last spoke? Why or why not?**
- [If did mention mental or physical health care at baseline] **Since we last spoke, how has the mental or physical care you received been?**
- **Regarding this health issue or condition, could you rate your current status? Rate your current status as: 1) very much improved, 2) much improved, 3) minimally improved, 4) no change, 5) minimally worse, 6) much worse, or 7) very much worse**
- Moral injury or stress: Are there any memories or experience from your (combat) deployment(s) or overall military service that continue to cause you stress and inner conflict since you’ve returned? How do these memories impact you and your reintegration process?

Critical Junctures (In health or reintegration experiences)

- Many people think about major milestones as typical parts of one’s life course. That can include finishing high school, starting a family, having a career, etc. Have there been any turning points or critical events that you can share?
  - How has your military experience defined who you are now?*
  - What has been most or least unexpected about your transition?*
- **We didn’t discuss much last time about aspects of your experience that might affect your transition. Do you think that gender shapes your experience in terms of reintegration? Does your race or ethnicity? Other forms of identity?**

Care Partner & Social Support

- **How have your experiences been with your family, friends, colleagues, and other civilians since you returned? How have your relationships changed? How do they contribute to your reintegration experience? Has anyone pointed out any changes in your behavior since we last spoke?**
- [If doesn’t have a care partner] Are there people or resources you’ve found helpful in the past 6 months? How have they helped you? For this question, you might think about the sociogram you drew for us at your first interview.
- [If has a care partner] Can you describe your relationship with your care partner? How has this person impacted you throughout your service, separation, and reintegration processes?

Military Experience and Veteran Service Organizations

- Can you describe in more detail your experience in the military, including your deployments, your perspectives on leadership, and other relationships with members of your unit?
- **During your transition out of military service, did you have a mentor or sponsor? If so, how was this person helpful? Was there another person who you consider to be an important influence?**
- Have you been part of any veteran or military service-related organizations? Why or why not? Do you prefer those that focus more on Post 9/11 veterans?

Next Steps

- **What advice would you give to other Veterans with similar experience?**
- **What are some of your expectations or goals for the next few years?**
- Do you have suggestions in terms of improving services for returning Veterans? What services or interventions are still needed? What are some services you’ve recently utilized that have been most helpful during your reintegration in the past 6 months?
- Is there anything you would like to add that we haven’t spoken about so far?

**VETERAN 24-MONTH INTERVIEW GUIDE**

| Participant ID: | Date: | Time: |
| --- | --- | --- |
| Interviewer(s): | | |

**Part B.1**

- Let’s start by touching base about your reintegration experience. You probably have some updates from the last time we spoke. Can you describe how things are going with respect to reintegration or adjustment to civilian life?
  - Have there been any changes with: your housing, job, or family situation?
- Can you walk me through a typical day that you’ve had in the last few months? What is that like for you, and what is different compared to six or 12 months ago? What is your day-to-day experience like?
- In thinking about health issues, physical or mental, can you talk about how they have played a role in your daily experiences since we last spoke?

**Part B.2**

Reintegration Experiences

- **Thinking about the last six months, have there been any changes in your expectations or goals? How are these expectations or goals being met? What about since we met a year ago?**
- **Do you feel more like a civilian now compared to when you first separated? How do you define reintegration and how has your definition of reintegration changed since you separated?**
- Would you say there has been any changes with regard to how you think about purpose or meaning in your broader life goals since your first interview?

Health Issues and Invisible Injuries

- **Have there been any changes with your health treatment or health services that you use since we last spoke?**
- **Do you see your condition getting better in the future? Do you have the resources you need to properly address your condition?**

Moral Injury

- One topic we may not have covered yet is moral injury. “Moral injury can occur in reaction to a traumatic event in which deeply held morals or values are violated. The resulting distress may lead to PTSD, depression, and other disorders in which feelings such as guilt, shame, betrayal, and anger are predominant.”
- Does this definition fit with your experience? Can you describe whether the issue of moral injury is something that has caused you stress and inner conflict in the last few years?

Military Deployments

- We didn’t have a chance to cover some details about your military career and deployment. Can you expand on [your deployment/aspect of military career)?
- Burn pits have been a common waste disposal practice, and emissions may have health effects. Are you aware of your own exposure to burn pits or other potentially toxic emissions? (Other common categories include (a) sand, dust, and particulate matter, (b) fuel, aircraft exhaust, and other mechanical fumes, (c) smoke from oil well fires, (d) last or noise injuries.)
  - Do you believe your potential exposures have affected you mentally or physically during your reintegration?
  - Have you enrolled or would you consider enrolling in the airborne hazards and burn pit registry?
  - Were you made aware of potential risks associated with exposure to burn pits during or after deployment? Were you ever provided any personal protective equipment when working around burn pits?
  - If treatments or screening tests became available for complications arising from toxic exposures, would you seek them out?

Social Difference

- Do you think that gender shapes your experience in terms of reintegration? Does your race or ethnicity? Other forms of identity?
- Is your experience with (this issue) during your reintegration similar to your experience in the military?

Critical Junctures (In health or reintegration experiences)**:**

- **Have there been any turning points or critical events that you can share?**
- How has your military experience defined who you are now?*
- If you haven’t already, can you describe your reasons for joining the military? What was your life like prior to joining? What were some of your expectations or hopes for joining?

Care Partner & Social Support

- **How have your experiences been with your family, friends, colleagues, and other civilians in the past 6 months? How have your relationships changed? How do they contribute to your reintegration experience?**
- [If doesn’t have a care partner] Are there people or resources you’ve found helpful in the past 6 months? How have they helped you? For this question, you might think about the sociogram you drew for us at your first interview.
- [If has a care partner] Can you describe your relationship with your care partner in the past 6 months? How does your relationship now compare to when we first talked with you? How has this person impacted you throughout your service, separation, and reintegration processes?
- [For parents] What has been most challenging about your experience as a parent while also reintegrating? What are some resources you wished you had?

Interventions

- **Fact or fiction: “The VA is doing better in terms of how it supports veterans with invisible injuries who have recently separated.” Based on your experiences, do you think that this is this fact or fiction, and why?**
- **What do you think is the #1 priority for the VA when helping veterans with invisible injuries who separated less than 5 years ago?**
- Do you have suggestions in terms of improving services for returning Veterans? What services or interventions are still needed? What are some services you’ve recently utilized that have been most helpful during your reintegration in the past 6 months?

This study

- **Why did you or did you not choose a care partner? Is there someone you would now recommend?**
- Do you consider this study itself as a sort of program that affects your experiences with adjustment or reintegration? Has this study caused you to reflect more on your reintegration experience and as a result, caused you to take specific steps towards your goals to better reintegrate?

Next Steps

- **Where do you see yourself in 5 years?**
- **What advice would you give to other Veterans with similar experience?**
- Is there anything you would like to add that we haven’t spoken about so far?
